# Seasonal Distribution, Composition, and Inventory of Plastic Debris on the Yugang Park Beach in Zhanjiang Bay, South China Sea

**DOI:** 10.3390/ijerph19084886

**Published:** 2022-04-17

**Authors:** Peng Zhang, Shanshan Wei, Jibiao Zhang, Huifeng Zhong, Shujia Wang, Qiying Jian

**Affiliations:** College of Chemistry and Environmental Science, Guangdong Ocean University, Zhanjiang 524088, China; zhangpeng@gdou.edu.cn (P.Z.); weishanshan1@stu.gdou.edu.cn (Shanshan Wei); zhonghuifeng11@stu.gdou.edu.cn (H.Z.); wangshujia@stu.gdou.edu.cn (Shujia Wang); jianqiying@stu.gdou.edu.cn (Q.J.)

**Keywords:** marine beach, plastic debris, seasonal distribution, tidal zone, composition, inventory

## Abstract

Plastic debris contamination in marine environments is a global problem that poses a considerable threat to the sustainability and health of coastal ecosystems. Marine beaches, as the key zones where terrestrial plastic debris reach coastal waters, are faced with the increasing pressures of human activities. In this study, we explored the distribution, composition, and inventory of plastic debris over seasonal and tidal zones at the Yugang Park Beach (YPB) in Zhanjiang Bay, South China Sea, to provide a baseline for plastic debris on a marine beach. The results showed mean abundance of plastic debris in summer (6.00 ± 2.10 items/m^2^) was significantly greater than that in winter (3.75 ± 2.12 items/m^2^). In addition, the composition of plastic debris ranged in size mainly from 1 to 5 mm and 0.5 to 2.5 cm in winter and summer, respectively. In terms of composition, white plastic debris was the most common (81.1%), and foam was the most abundant (64.4%). Moreover, there was a significant relationship between the abundance of plastic debris and sand grain size fraction (*p* < 0.05), implying the abundances of microplastic debris were more easily impacted by sand grain size (>2 mm). In total inventory, there were about 1.18 × 10^5^ and 2.95 × 10^5^ items of plastic debris on the YPB in winter and summer, respectively. The tidal variation and human activities are responsible for the plastic debris accumulation. This study provided a method to quantify the inventory of plastic debris on a beach and could be helpful to consider regional tidal variations and critical source areas for effective plastic debris clean-up.

## 1. Introduction

The presence of plastic debris in the environment is a result of global production and the uncontrolled discard of plastic products [1,2,3]. The COVID-19 pandemic has especially reemphasized the indispensable role of plastics in our daily life [4,5]. Global simulations of marine plastic transport have shown plastic debris trapping in coastal zones [6]. Moreover, plastic debris can also be found on beaches in the Arctic [7]. Globally, approximately 80% of anthropogenic marine plastic debris is derived from land-based sources [3,8]. Plastic pollution can be divided into two broad categories: macroplastic pollution and microplastic pollution [9,10]. At present, plastic particles smaller than 5 mm are usually called microplastics [11,12]. One category of microplastics, which comes from the wave breaking of large plastic debris in the environment via solar radiation or wave cracking, is known as ‘secondary’ microplastics [13]. Another type is directly produced at micro sizes; these are known as ‘primary’ microplastics [14]. Poorly managed plastic waste in the ocean, including plastic debris and its fragments, can affect water quality and pose a threat to marine ecosystems [15,16]. In addition, plastic debris circulates in the environment and breaks into smaller pieces of various sizes [17,18]. A particular concern is the occurrence of smaller pieces of plastic debris, including those not visible to the naked eye [19,20]. Consequently, the tiny plastic particles can also be mistaken for food by invertebrates, turtles, fish, seabirds, and large marine mammals [21,22]. Due to different physical mechanisms of transport, in particular, currents and tides, plastics are widespread in all marine environments [23].

Macroplastics and microplastics can accumulate on beaches worldwide [24,25,26]. Marine beaches, as the key zones where land-based plastic debris reach coastal waters, are faced with the increasing impacts of human activities and natural tidal variation [27,28,29]. Previous studies have explored the abundance and geographic distribution of anthropogenic plastic debris on beaches and in marine environments, such as estuaries, bathing beaches, and the beaches impacted by intensive human activities [30,31,32]. Global plastic debris monitoring of beaches plays important roles in quantities, composition, and sources of plastic debris [33,34,35]. In addition, the different sizes of plastic debris have been found and identified in beach sediment, such as on the Algerian western coast [36], urbanized beaches (Da Nang, Vietnam) [37], and beaches around the northern South China Sea [38]. Plastic debris on sandy beaches with different human uses and waste management have different sources and spatiotemporal variation [39,40]. Land-based plastic waste is a key source of coastal pollution from beaches [17,41]. The presence of large quantities of plastics on beaches has a negative impact on tourism and the cost of ongoing cleaning operations [42,43]. Therefore, the strategy for mitigation of plastic debris should be to explore the sources and composition [44,45]. In addition, due to the complex interaction between plastic debris and toxic chemicals, the toxic chemicals can be transported and carried by plastic debris [46,47]. Moreover, the microplastics on beaches can also introduce toxic chemical substances to reduce system functions linked to health and biodiversity [48,49]. Furthermore, the combination of high levels of ultraviolet (UV) radiation and mechanical abrasion makes marine beaches hotspots for microplastic formation [50,51], and once macroplastics break down into microplastics, they are much more difficult to manage [51]. The empirical data for macroplastics was less robust than for micro-plastics [52]. Thus, it is necessary to explore the relationships between the differences in plastic debris sizes. Understanding this relationship could be useful for collecting information on the distribution and abundance of microplastics. However, previous studies have shown the abundance of plastic debris to be easily impacted by anthropogenic activities, seasonal and tidal variation, and behaving markedly different [53,54,55]. For example, if the sampling stations are located in the high tidal line of a marine beach, the abundance of plastic debris may be overestimated [41]. Thus, the field sampling stations and time play a key role in the abundance of plastic debris, which should consider the tidal and seasonal change. Moreover, although a few studies have examined the size-dependent abundance of plastic debris [56,57], there is currently little work on relationships between the total amount of plastic debris of different sizes and their interaction with sand sediment sizes [58,59]. In particular, the microplastics may be easily impacted by the larger sand sediment size. The concurrent investigation of both macro- and microplastics can help us fully explore the sources and composition of plastic debris.

**Figure 1:**
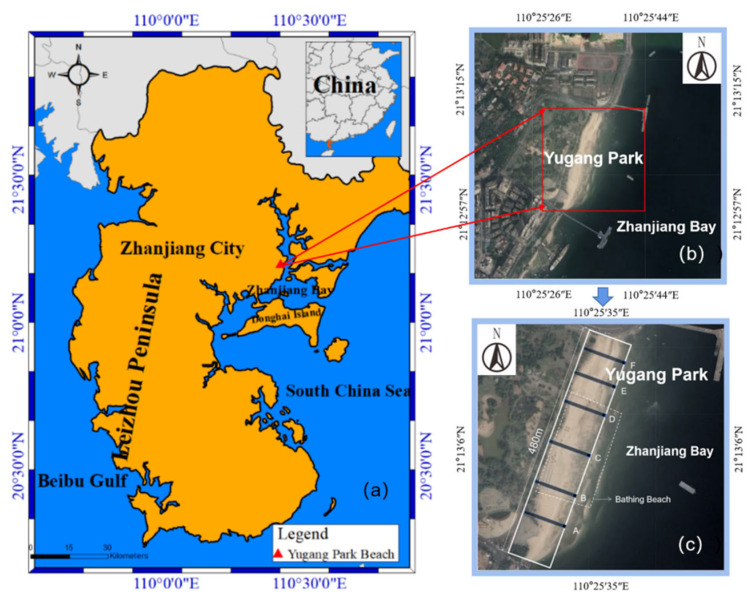
Geographical location of Zhanjiang Bay (**a**), Yugang Park (**b**), and sampling sections (**c**).

Zhanjiang Bay (ZJB) is a subtropical semi-enclosed bay on the Leizhou Peninsula (Figure 1a), South China Sea, on the southernmost part of the Chinese mainland [60,61]. It is mainly dominated by the irregular semidiurnal tides [61]. The difference in the annual average tidal range between Tiaoshun Island and the bay mouth is about 0.6 m and the annual maximum tidal range difference is 0.8 m [62,63]. The Yugang Park Beach (YPB) is on the northern end of the sea observation corridor in the center of ZJB, covering an area of approximately 200,000 m^2^ (Figure 1b). It opened in 2013 after a makeover. Due to a large number of tourists who visit the YPB every year, plastic debris produced from land-based sources is not easy to completely clean up; the high reliance on tourists’ self-awareness results in the accumulation of plastic debris on the beach. In addition, changes in tides and coastal currents during high or low tides may move debris from the beach down the fault plane and leave plastic debris from the ocean on the beach. In addition, plastic debris can also migrate along the surface of the beach because of the effect of the tropical monsoon climate [53,54]. There have been studies in the past on how land-based sources affect eutrophication and degrades water quality [61,64]. In addition, ZJB is also currently a large site for marine aquaculture on the coast [58]. High-intensity aquaculture activities in the ZJB have a significant impact on the coastal environment [61,65]. A previous study found microplastics in 30 out of 32 fish species at an average abundance of 2.83 ± 1.84 items individual^−1^ in Zhanjiang mangrove wetland [66]. However, there have been no studies on plastic debris enrichment on the YPB covering the tidal zones and seasonal variation. Thus, the plastic debris pollution poses a threat to the sustainable development and safety of marine food products and to human health.

Therefore, the 24 sand sediment samples covering the dry beach and high, middle, and low tide zones on the YPB were collected via field sampling in January and July 2021, respectively. The objectives of this study were to (1) investigate the spatial pattern of plastic debris at the YPB, (2) determine the characteristics of plastic debris composition, (3) analyze the relationships between the abundance of plastic debris and sand fraction, and (4) quantify the inventory of plastic debris on the YPB. This study can provide a method to quantify the inventory of plastic debris on a beach and could be helpful in the consideration of regional tidal variations and critical source areas for effective plastic debris clean-up around the word.

## 2. Materials and Methods

### 2.1. Study Area

The total length of ZJB is 54 km from south to north, and it is 24 km wide [64]. It is a semi-closed bay with weak hydrodynamic conditions covering an area of 193 km^2^ [61]. In addition, its deep channel (more than 10 m deep) is 40 km long with a mouth approximately 2 km wide. In recent years, the outlets of many municipal sewage treatment plants have been placed along the coast and a large amount of industrial wastewater is discharged into the bay, which have led to the degradation of water quality [60]. The YPB is on the western coast of ZJB; the beautiful environment of the latter as well as the availability of convenient transportation means that many tourists from Zhanjiang City are attracted to visiting the YPB for marine recreation every year (Figure 1b). This brings several economic benefits for the development of Zhanjiang City, but places considerable pressure on protecting the marine beach and coastal aquatic environments. Though there are regular clean-up efforts by workers every day, the microplastic debris can also be found in the YPB. Additionally, the lack of marine environmental awareness and education of recreational activities on the beach may pose a threat to the marine environment through plastic pollution.

### 2.2. Sampling and Analysis Method

**Figure 2:**
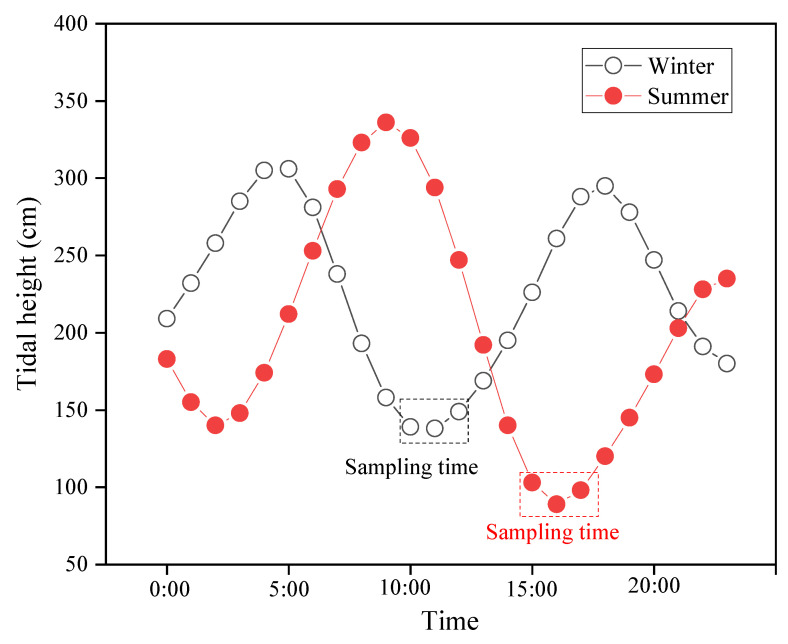
Seasonal variations in height for the tide in Zhanjiang Bay.

To understand the inventory, composition, and seasonal distribution of plastic debris on the YPB, we divided a section of the coastline into six sections (A, B, C, D, E, and F) (Figure 1c). To assure the accuracy and representativeness of different seasons, the field samplings were all performed during the low tidal height period on 7 January and 7 July 2021 (winter and summer, respectively) after the workers’ clean-up efforts. Each section contained four stations, covering dry beach and the high, middle, and low tide zones, for a total of 24 sampling stations (Figure 1c). The sampling stations and process were not impacted by the human recreational activities and ocean currents in each season. Procedures to sample sandy beaches using standardized methods to modify and provide detailed information have been previously published [41,67]. To assure complete sampling in a short time and to avoid sampling stations affected by the tidal vitiation (Figure 2), the sampling team was divided into six groups corresponding to the six sections on the YPB. First, sand samples were collected from the low tide line at the boundary between the beach and the water. Then, based on changes in tide, we identified and sampled from the high tide line in the high tide zone and the middle tide zone (between the low and high tide lines). Finally, dry sand samples were collected from the dry beach zone between the backshore and high tide lines.

We collected sand from the surface of the beach, i.e., depths up to 1 cm, in a sampling frame (0.5 m × 0.5 m) using a metal spoon. The spoons were washed with seawater prior to sampling. We then put the samples into labeled bags and quickly transported them to the laboratory after sampling was completed [68]. Finally, 48 surface sand samples were obtained for further analysis. In this study, the range of sizes for microplastics and ‘mesoplastics’ were limited to 1–5 mm and 5–25 mm, respectively. ‘Macroplastics’ (>2.5 cm) were further divided into those sized 2.5–5 cm, 5–10 cm, and >10 cm [41].

In the laboratory, the sand samples were first homogenized in aluminum trays and then dried at 60 °C for 24 h (Figure S1). To avoid background contamination, samples were covered with aluminum foil. The dried samples were then stored at 25 °C in sealed glass bottles until extraction [55,69]. Plastic tools or containers were not used during sampling or laboratory analysis to avoid additional plastic contamination. Plastic particles were extracted using the density separation method, with saturated NaCl solution (density 1.2 g/cm^3^) as the density liquid [41]. After precipitates settled, the supernatant was passed through a 1 mm mesh sieve. This process was repeated at least five times until no visible particles were observed in the supernatant. The particles collected in the sieve were washed and the residue on the sieve was transferred to a 250 mL beaker. To dissolve the natural organic matter in the sand sample, 20 mL of 30% H_2_O_2_ and 20 mL of 0.05 M Fe(II) solution were added to the beaker containing the sample. The beaker was then heated on a hot plate to 75 °C for 12 h and cooled at room temperature for 24 h. Finally, the suspects were transferred to small glass vials with ultra-pure water to remove salt and put through a second drying step. The separation and identification techniques are in accordance with the described protocols of analysis [41,69]. The color, shape, and abundance of all plastic debris (>5 mm) were counted on the filter membrane by visual inspection method, and the maximum length size of the irregular plastic debris was determined and measured by the ruler. Moreover, microplastics in the 1–5 mm size range were quantified by systematic counting under a stereo microscope (SMZ1270i, Nikon, Tokyo, Japan) of up to 40 × 40 magnification [69]. Fourier transform infrared spectroscopy (Bruker, OPTIK GMBH, Karlsruhe, Germany) was used to determine the plastic debris composition. In addition, sand grain size analysis was performed according to the Specifications for Oceanographic Survey Part 8: Marine Geology and Geophysics Survey (GB12763.8-2007) [41,70].

### 2.3. Data Analysis

A mathematical statistical analysis of the abundance of plastic debris at the 24 sampling stations was used to quantify the seasonal total inventory of plastic debris present on the YPB. The beach was divided into 24 small blocks using Google Maps, with each sampling station used as the central point of the small block. Thus, the total inventory of plastic debris on the YPB in each season can be estimated using Equation (1) below:

(1) $$N=\sum_{i=1}^{24}(S_{i}•n_{i})$$

where *S_i_* is the individual area of the small block on the YPB, of which the sampling station is the center (m^2^), *n_i_* is the number of items of plastic debris at each sampling point (items/m^2^), and N represents the total amount of plastic debris on the YPB (items).

### 2.4. Statistical Analysis

Microsoft Excel 2013 was used to analyze plastic debris data and graphs were generated using the software Origin2021. The one-way analysis of variance (ANOVA) and Tukey’s Honest Significant Difference (HSD) tests were used to determine spatial tidal zones and seasonal differences in SPSS 22; all correlation analyses were determined to be significant at *p* < 0.05. A map of the station locations was drawn using Google Earth and ArcGIS 10.2.

## 3. Results and Discussion

### 3.1. Seasonal Pattern of Plastic Debris on the YPB

**Figure 3:**
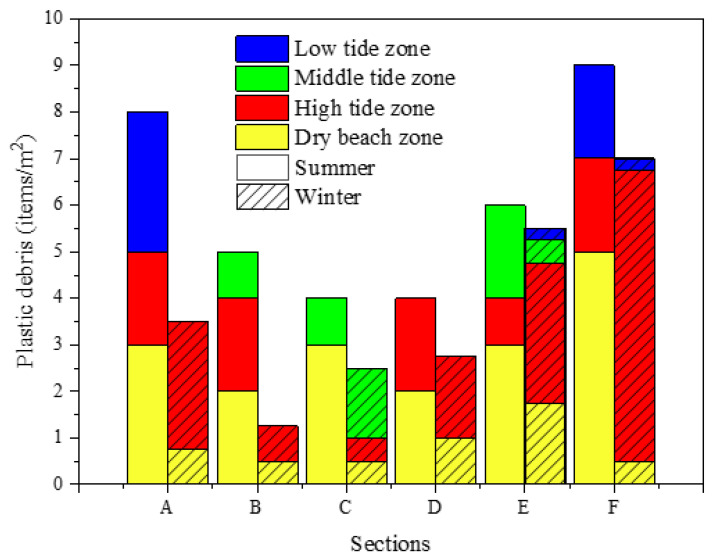
Seasonal distribution of plastic debris on the Yugang Park Beach.

The seasonal distribution of plastic debris on the YPB is shown in Figure 3. The mean abundance of plastic debris in summer (6.00 ± 2.10 items/m^2^) was significantly greater (1.6 times) than that in winter (3.75 ± 2.12 items/m^2^). The main reason was caused by the frequent human recreation activities in summer season. A total of 90 plastic debris items were detected in the sand samples in winter. In addition, sections A, B, C, D, E, and F contained 15.6%, 5.6%, 11.1%, 12.2%, 24.4%, and 31.1% of plastic debris, respectively. In terms of spatial distribution, the smallest abundance of plastic debris identified included only two items in the low tide zone. However, the highest amount detected was 60 items in the high tide zone. In summer, a total of 36 items of plastic debris were found in the various study sections. In addition, sections A, B, C, D, E, and F contained 22.2%, 13.9%, 11.1%, 11.1%, 16.7%, and 25%, respectively, of the plastic debris. The mean abundance of plastic debris in the tidal zone was significantly different according to the ANOVA and Tukey’s HSD tests (*p* < 0.05). The highest abundance of plastic debris, accounting for 50% of the total, was found in the dry beach zone; in the high and low tidal zones these values were 25% and 13.9%, respectively. The lowest abundance of plastic debris, 11.1%, was in the mid-tide zone. In both seasons, section F accumulated the most plastic debris, with the majority of this coming from the dry beach and high tide zone.The marine beach zone is the main area where plastic debris accumulates [26]. There was significant seasonal variation in the plastic debris on the YPB. The greatest amount of plastic debris was found in the high tide zone, followed by the dry beach, middle tide, and low tide zones. Plastic debris particles were only found in the low tide zones of sections E and F, in particular, while other samples were not found there. Most of the plastic debris imported from terrestrial sources remained on the beach, while some plastic debris may have migrated from the low and middle tide zones and seawater to the high tide zone and dry beach as the tides changed. Therefore, most of the plastic debris on the YPB likely accumulated in high tide zones and dry beaches due to a lack of dynamic tidal power [71,72].

Beaches are links between the land and the sea, and sourcing plastic debris found on beaches is relatively complex [45]. From transport boundaries, sources include terrestrial pollution, pollution carried in seawater, and the air [9,73]. Human activities related to aquaculture, particularly wastewater discharge, are the largest sources of pollution in ZJB [60]. In addition, large quantities of sewage discharge and piles of garbage are important sources of plastic debris from terrestrial sources, as are anthropogenic waste and living plastic waste and some landfill or sewage treatment plant waste generated from surface runoff or estuarine inputs onto beaches or into coastal water [74,75,76,77]. Land-based plastic waste on the YPB is also inseparable from human tourism activities and domestic plastic waste sources. Marine pollution sources include shipping and aquaculture activities [58,78]. At the same time, regional changes in climate such as rainfall and typhoons have also impacted plastic debris distribution patterns [53,54]. Some plastic debris is transported to beaches by heavy winds and rainfall-induced stormwater runoff. In addition, riverine flows also increase sharply during the summer rainfall season. Large items of plastic debris may be cracked and weathered to form microplastics [79]. The distribution of plastic debris on the YPB was also affected by beach bathing. The bathing area at the YPB is located above the mid-tide line between sections B, C, and D. The average number of plastic debris items in this area was 2.17 ± 0.80 items/m^2^ in winter and 4.33 ± 0.58 items/m^2^ in summer. The average number of plastic debris items in the non-bathing area was 5.33 ± 1.76 items/m^2^ in winter and 7.67 ± 1.53 items/m^2^ in summer. This may be because the safety net in the bathing area gathered a greater part of the macroplastics present than the mesh aperture in the coastal water, leading to a decrease in the abundance of plastic debris in the sand sample [80].

Compared with other beaches worldwide, the YPB has a relatively low level of plastic debris pollution (Table 1). The mean number of items of plastic debris on the YPB is lower than that on the Baltic beaches of Russia; the continental coast of Chile; Lover Beach, Zhuhai; Sanniang Bay, Qinzhou; Shiluo Kou, Weizhou; Black Sand Beach, Macau; and Gaviotas, Socorro, Cristianos, and Arena, Spain [18,81,82,83]. However, the abundance of plastic at the YPB was higher than on Tejita Beach in Spain [83] and the pollution level was almost the same as on Cheung Sha Beach, Hong Kong [82]. The levels of plastic debris pollution on different beaches can reflect regional socioeconomic development and plastic waste management policies. The mean abundance of plastic debris on the First Long Beach, which is adjacent to land-based sources in Zhanjiang City, was 10 times higher than the mean abundance of plastic debris on the YPB; this indicates the great impact of land-based sources of plastic debris [41]. In addition, the variation in the abundance of plastic debris is significant, possibly due to the different impacts of human activities and changes in the marine environment [53,54,84,85].

### 3.2. Seasonal Size, Shape, and Color Composition of Plastic Debris on the YPB

**Figure 4:**
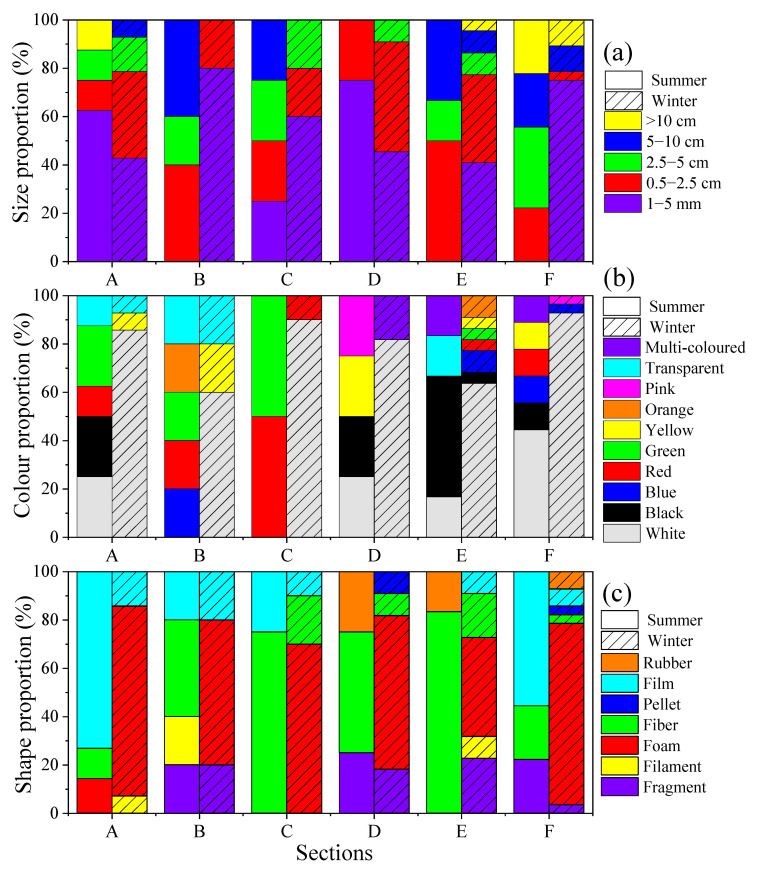
Seasonal size proportion (**a**), colour proportion (**b**), and shape proportion(**c**) of plastic debris on the YPB.

As noted earlier, the plastic debris samples collected were divided into the following five categories based on their size: 1–5 mm, 0.5–2.5 cm, 2.5–5 cm, 5–10 cm, and >10 cm. There was more plastic debris, except for microplastics (1–5 mm) in summer than winter (Figure 4a). Results for winter showed that the greatest amount of microplastics present accounted for 56.7% of the total and that most of these were found in section F. Plastic debris larger than 10 cm was the lowest fraction at only 4.4%, and were only found in sections E and F (Figure 4a). Therefore, the smaller the particle size the higher its abundance in winter. Plastic debris in the 0.5–2.5 cm, 2.5–5.0 cm, and 5–10 cm fractions accounted for 24.4%, 7.8%, and 6.7%, respectively. In summer, plastic debris 0.5–2.5 cm in size was the most abundant and existed in all six sections, accounting for 27.9%. The second most abundant size of debris was microplastics, which accounted for 25% of the total. The most microplastics were detected in section A. The plastic debris sized 2 cm and 5–10 cm had the same abundance, accounting for 19.4%. Plastic debris in fractions larger than 10 cm accounted for the smallest part of the total at 8.3%, only found in sections A and F.

Additionally, the investigation showed that plastic debris were identified in a total of 10 colors (Figure 4b). Among the 90 items of plastic debris collected in winter, the main color was white (81.1%). The greatest abundance of white plastic was found in section F followed by sections E and A. Other colors were present as less than 5% of the samples. Items colored black, green, and pink were the least commonly found; they accounted for only 1.1% of the plastic debris. In summer, white and black debris were the most abundant and second most abundant colors in sand samples at 22.2% and 19.3%, respectively. White plastics were most common in section F and black plastics were most common in section E. The other colors accounted for less than 15% of the total. Orange and pink debris made up the smallest proportions of the total, at only 2.9%. Orange only exists in section B, pink only exists in section D. In the plastic samples in winter and summer, white was the most abundant. In all sections, there were more of all colors except white in summer than in winter.

**Figure 5:**
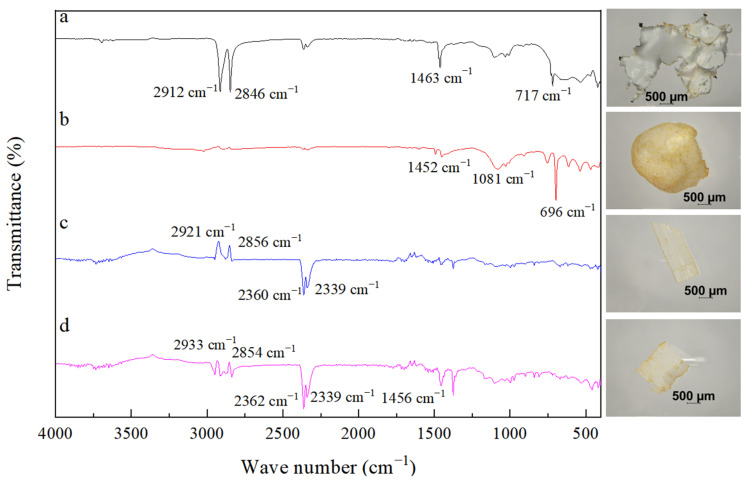
Typical identified microplastics and their compositions. Polyethylene (**a**), polystyrene (**b**), polyvinyl chloride resin (**c**), polypropylene (**d**).

Moreover, the results of the investigation showed that foam plastics (64.4%) were dominant in the winter samples, particularly from section F (23.3% of the total) (Figure 4c). This was followed by fragments (10%). Rubber and pellet shapes were the least (2.2%) and only appeared in sections D and F. Plastic fragments appeared in sections A and E, accounting for 3.3% of the total plastic. Plastic film appeared in all sections except section D and accounted for 8.9% of the total. Fibers were found in sections C, D, E, and F, also accounting for 8.9% of the total. The most plastic shapes, six, were detected in section F; this was followed by section E, in which five shapes were detected. The most abundant plastic materials in the samples differed in winter and summer; fiber (41.7%) and film (36.1%) were the most common in summer. There was a fragment shape (11.1%). Rubber (5.6%) appeared in sections D and E. Filaments (2.8%) only appeared in section B while foam (2.8%) only appeared in section A. No pellet plastic was found in summer. Different main polymers were found in selected samples of white film (A), white foam turning yellow (B), transparent fragments (C) and transparent fibers (D), with the main types including polyethylene (a), polystyrene (b), polyvinyl chloride resin (c), and polypropylene (d) (Figure 5).

In terms of the composition of the plastic debris, results showed that microplastics (1–5 mm) were most abundant in winter (accounting for 56.7% of the total). In contrast, the number of plastic debris sized 0.5–2.5 cm was highest in summer, accounting for 27.9% of the total. The change in the dominant size indicated that the composition of plastic debris showed seasonal variation. The results for winter were consistent with those from a previous study on the Beihai Silver Beach [82]. Furthermore, the likelihood that marine organisms will ingest microplastics increases as the size of the microplastics decreases [86]. In addition, the 10 colors of plastic debris identified in the YPB indicated the complexity of their sources. White plastic debris was predominant among the plastic debris from the YPB; future research could focus on the chemical composition of white plastic debris to explore its toxicity for marine biology. Foam, fiber, and film were the dominant debris materials in the YPB, indicating that local fishery and tourism at beaches along ZJB contributed to their deposition.

### 3.3. Interactions between Plastic Debris and Sand Sediment on the YPB

**Figure 6:**
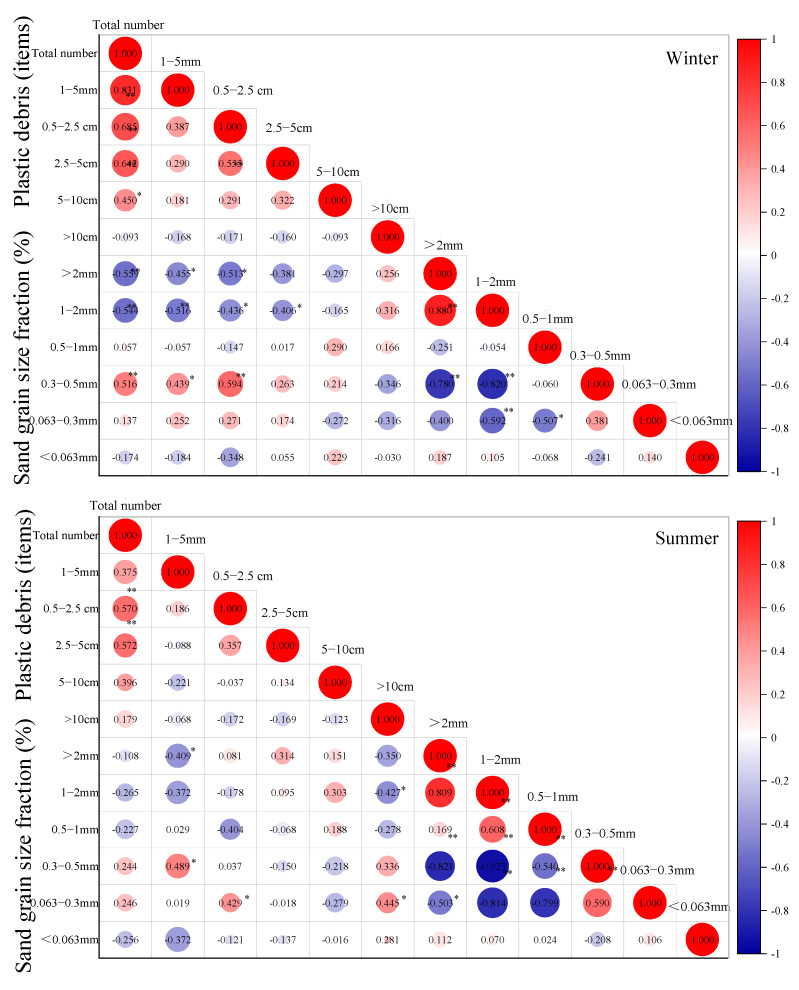
Spearman correlation coefficients between the items of plastic debris by size and sand grain size fraction (*n* = 24). Note: * refers to correlations significant at *p* < 0.05 (two-tailed) and ** refers to correlations significant at *p* < 0.01 (two-tailed).

The correlation between the abundance of plastic debris and sand size fraction on the YPB is shown in Figure 6. The results revealed that the total abundance of plastic debris was significantly positively correlated (*p* < 0.05) with their different sizes; the exception was plastic debris >10 cm in winter. In winter, the total abundance of plastic debris had a higher correlation (0.831, *p* < 0.01) with microplastics than with mesoplastics (0.685, *p* < 0.01). In addition, the abundance of plastic debris sized 2.5 cm in winter was positively correlated with the abundance of plastic debris at size 0.5–2.5 cm (0.555, *p* < 0.01), indicating that mesoplastics came from the same sources in winter [41]. In contrast, the total abundance of plastic debris had significant positive correlations with mesoplastics at sizes 0.5–2.5 cm (0.570, *p* < 0.01) and 2.5–5.0 cm (0.572, *p* < 0.01) in summer.

Furthermore, there were significant relationships between the abundance of plastic debris and sand grain size fractions (Figure 5). In winter, the total abundance of plastic debris was significantly negatively correlated with some sand grain size fractions (>2 mm = −0.559, *p* < 0.01; 1–2 mm = −0.544, *p* < 0.01) and positively correlated with others (sand grains 0.3–0.5 mm = 0.516, *p* < 0.01). In addition, the abundance of plastic debris (1–5 mm and 0.5–2.5 cm) also had significant relationships with sand grain size fraction (>2 mm, 1–2 mm, and 0.3–0.5 mm). In contrast, there was no significant correlation between the total abundance of plastic debris and the sand grain size fraction. However, the abundance of microplastic debris (1–5 mm) was negatively correlated with the sand grain size fraction >2 mm (−0.409, *p* < 0.01) and positively correlated with the sand grain size fraction 0.3–0.5 mm (0.489, *p* < 0.01). This revealed that the abundance of microplastic debris was more easily impacted by sand grains sized >2 mm, compared to mesoplastic and microplastic debris, on the YPB. A previous study indicated that microplastic debris can be retained at the interface between sediment layers of different grain and pore sizes [87]. Moreover, the sand grain size fraction that was similar in size to the microplastic debris (>2 mm and 1–2 mm) had a significantly negative relationship with the sand grain size (0.3–0.5 mm). Sand grains sized >1 mm could possibly have broken down into smaller sizes under tidal dynamics and thus allowed microplastic debris to return to the sand.

### 3.4. Quantifying the Inventory of Plastic Debris on the YPB

**Figure 7:**
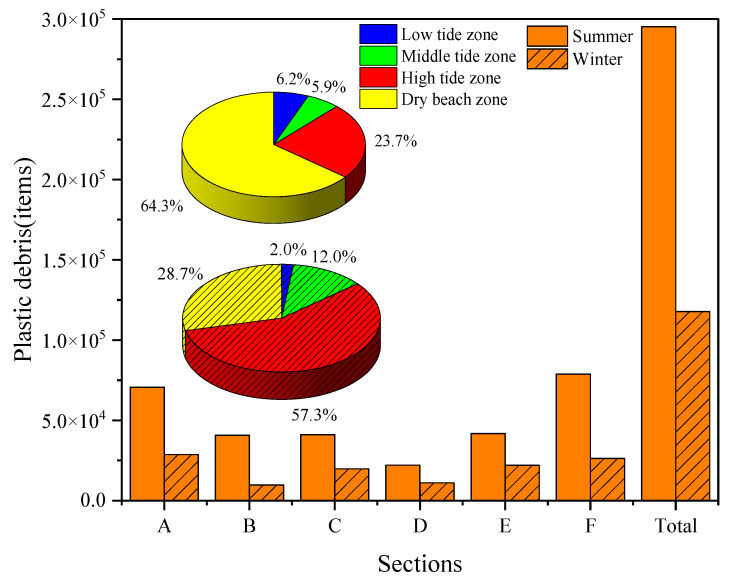
The inventory of plastic debris on the Yugang Park Beach.

Based on the individual area of each small block, the inventory of plastic debris present on the YPB in each season was quantified using Equation (1). A total of 1.18 × 10^5^ and 2.95 × 10^5^ items of plastic debris were estimated to have been present in winter and summer, respectively (Figure 7). There were marked variations in the types of debris present in the different sections. In winter, the A section had the highest proportion of debris (24.3%), which was 28,665 items. However, section B had the lowest number (9744) of items. In summer, the highest proportion of debris was found in section F (26.7%); 78,840 items of plastic debris accounted for this. Section D contained the least number of items and accounted for 7.5% of the total. The proportion of plastic debris in the tidal zone was significantly different (*p* < 0.01) from the rest. In winter, the proportion of plastic debris in the high tide zone was the highest (57.3%) at 67,467 items; this was followed by the dry beach zone (28.7%) with 33,824 items and the low tide zone with the lowest amount of plastic debris 2.0%, or only 2296 items. In summer, plastic debris accounted for the highest proportion (64.3%) of the dry beach zone. The second greatest proportion of plastic debris was in the high tide zone (23.7%), with 69,872 items. The amount of plastic in the low and middle tide areas was relatively low, accounting for only 6.2% and 5.9% of the total, respectively.

The YPB is a tourist beach, and tourist activities thereon may be a direct source of plastic debris [84,88]. In addition, different sizes of plastic debris have an impact on the difficulty of cleaning marine beaches, with the spatial distribution thereof also having implications for this [41,44]. The seasonal distribution of plastic debris showed a disproportional total amount on the YPB (Figure 7). The abundance of plastic debris in the high tide and dry beach zones of sections A, B, E, and F increased significantly in winter; the number of items of plastic debris was high in these areas, for which the critical source areas should be given more attention during clean-up attempts [41]. Although tourism activities at the YPB decline in winter, plastic debris remaining on the beach can be broken down into smaller and more toxic microplastics at any time. In addition, microplastic debris can be retained at the interface between sediment layers of different grain and pore sizes [87]. Therefore, cleaning up plastic debris on beaches should focus on smaller items to reduce the amount of secondary microplastics generated and the risks from plastic debris flux in the coastal water. In comparison, the abundance of plastic debris on the YPB was relatively high in summer, particularly in the high tide and dry beach zones. This was due to the large number of tourist activities, such as bathing, eating, and drinking, occurring on the beach at that time. In addition, the number of items of microplastic was lower than other sizes of plastic debris (0.5–2.5 cm) in summer (Figure 4). On the one hand, microplastics can easily be transported by coastal water given tidal dynamics [87]. On the other hand, plastic debris <1 mm in size were not considered in this study, leading to a lower abundance of microplastic debris in sand samples. At the same time, medium-sized plastics are not always removed by cleaners in a timely manner, and large items of plastic debris on the beach can be ground into smaller fragments by human activity and increase the concentration of microplastics in surface sand layers [41]. This could also increase risks from smaller and more toxic microplastics. At the same time, the plastic on beaches, which may have been generated by human activity, comes in diverse compositions. For example, more than 64.4% of the plastics identified in the samples were foam; its particles have a common shape, small size, and are often overlooked during cleaning activities. This means that they accumulate easily on beaches. Furthermore, marine aquaculture is highly developed in ZJB, and foam materials are widely used in the storage and ship of fish and shrimp. Advanced cleaning technologies should be developed to reduce foam pollution in the YPB. The sources of the white colored plastic debris should be traced and recycled.

## 4. Conclusions

In summary, this study observed significant seasonal variations in the distribution of plastic debris on the beaches of YPB and revealed the importance of tide and human activities in shaping the inventory of marine plastic debris. The abundance of plastic debris on the YPB in summer (average: 6.00 ± 2.10 items/m^2^) was significantly higher than in winter (average: 3.75 ± 2.12 items/m^2^, *p* < 0.05), which may be caused by frequent human activities. In addition, the plastic debris detected on the YPB ranged mainly from 1 to 5 mm and 0.5 to 2.5 cm in winter and summer, respectively. In terms of the composition of the plastic debris, white was the most common color, and foam was determined to be the most abundant (64.4%), which provide the key information of tracing the plastic debris sources in future. Moreover, a total plastic debris of 1.18 × 10^5^ and 2.95 × 10^5^ items was estimated to be present on the YPB in winter and summer, respectively. Plastic debris hotspots were identified in the high tide and dry beach zones in winter and summer, respectively. Thus, field sampling of plastic debris should consider the impacts of tidal and seasonal variation in the future. These results suggest that exposure to human activities and tides from the ZJB may be a major determinant of plastic debris disproportional accumulation on tidal zones. This study provided the inventory of plastic debris on a beach and presented a useful mitigation strategy for considering variations in regional tide time and critical source areas for marine beach clean-up efforts. To effectively reduce the amount of plastic debris on beaches, all sectors of society must make a joint effort to reduce inputs from anthropogenic sources, clean beaches regularly, and prevent plastic debris from entering the coastal waters. 

## Figures and Tables

**Table 1:** Comparison of the abundance of plastic debris on beaches worldwide.

Marine Beach	Size (mm)	Average Abundance (Items/m^2^)	References
Baltic beaches, Russia	0.5–5	42–1150	[18]
Continental coast of Chile	1–10	27.0	[81]
Lovers Beach, Zhuhai	1–20	34.70	[82]
Sanniang Bay, South China Sea	1–20	14.0	[82]
Shiluo Kou, Weizhou	1–20	178.0	[82]
Beihai Silver Beach	1–20	30.0	[82]
Cheung Sha Beach, Hong Kong	1–20	3.0	[82]
Black Sand Beach, Macau	1–20	13.0	[82]
Heungnam Beach, South Korea	>2	473 ± 866	[83]
Gaviotas, Spain	>2	11.68 ± 17.41	[83]
Tejita, Spain	>2	1.50 ± 5.69	[83]
Puertito, Spain	>2	162.71 ± 342.01	[83]
Cristianos, Spain	>2	12.38 ± 49.93	[83]
Arena, Spain	>2	10.47 ± 27.71	[83]
First Long Beach adjacent to land-based sources, South China Sea	>1	34.0	[41]
Yugang Park Beach, South China Sea	>1	4.88	This study

## Data Availability

Data sharing is not applicable for this article.

## Supplementary Materials

The following supporting information can be downloaded at: https://www.mdpi.com/article/10.3390/ijerph19084886/s1, Figure S1: Pretreatment steps for sand samples: (a) Weigh the sand samples; (b) Dry the samples at 60 °C for 24 h; (c) Add saline solution to the sediment and let stand until stratification occurs; (d) Pass supernatant through a 1 mm mesh sieve; (e) Add 20 mL of 30% H2O2 and 20 mL of 0.05 M Fe(II) solution to the beaker containing the sample to dissolve the natural organic matter; (f) Heat on a hot plate in a 75 °C water bath for 12 h and cool for 12 h; (g) Remove plastic >5 mm; (h) Carry out quantitative analysis for microplastics in the 1–5 mm range.
