# Supplementary material for: Seasonal Distribution, Composition, and Inventory of Plastic Debris on the Yugang Park Beach in Zhanjiang Bay, South China Sea
Source: Int J Environ Res Public Health. 2022 Apr 17;19(8):4886. doi: 10.3390/ijerph19084886 (PMC9032269; doi:10.3390/ijerph19084886)
Supplement: Supplementary file 1 [file ijerph-19-04886-s001.zip › ijerph-1632765-supplementary.pdf]

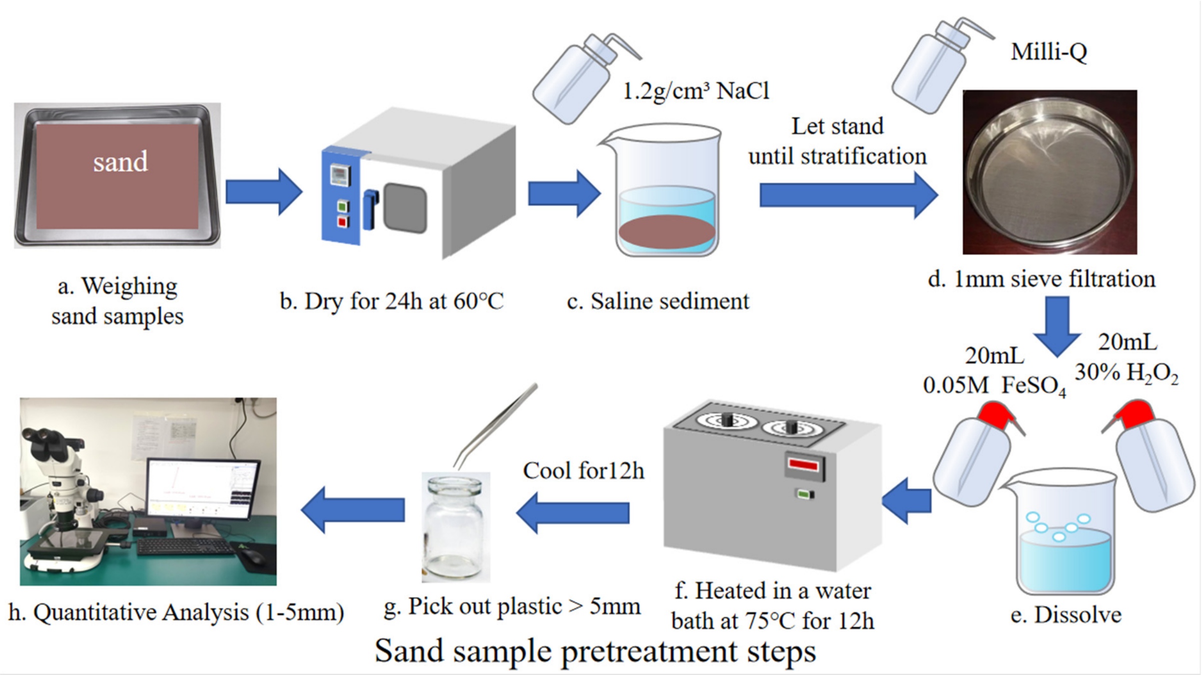

Figure S1. Pretreatment steps for sand samples: (a) Weigh the sand samples; (b) Dry the samples at 60 °C for 24 h; (c) Add saline solution to the sediment and let stand until stratification occurs; (d) Pass supernatant through a 1 mm mesh sieve; (e) Add 20 mL of 30% H_2O_2 and 20 mL of 0.05 M Fe(II) solution to the beaker containing the sample to dissolve the natural organic matter; (f) Heat on a hot plate in a 75 °C water bath for 12 h and cool for 12 h; (g) Remove plastic >5 mm; (h) Carry out quantitative analysis for microplastics in the 1–5 mm range.
